# The Structure of Metals

**Published:** 1881-05

**Authors:** 


### THE STRUCTURE OF METALS.

The following observations on the minute structure of metals, which have been hammered into thin leaves, are instructive. Notwithstanding the great opacity of metals, it is quite possible to procure, by chemical means, metallic leaves sufficiently thin to examine beneath the microscope by transmitted light. Silver leaf, for instance, when mounted upon a glass slip and immersed for a short time in a solution of potassium cyanide, perchloride of iron, or iron-alum, becomes reduced in thickness to any required extent. The structure of silver leaf may also be conveniently examined by converting it into a transparent salt by the action upon it of chlorine, iodine, or bromine. Similar suitable means may also be found for rendering more or less transparent most of the other metals which can be obtained in leaf.

An examination of such metallic sections will show two principal types of structure, one being essentially granular, and the other fibrous.

The granular metals, of which tin may be taken as an example, present the appearance of exceedingly minute grains, each one being perfectly isolated from its neighbors by still smaller interspaces. The cohesion of such leaves is very small.

The fibrous metals, on the other hand, such as silver and gold, have a very marked structure. Silver, especially, has the appearance of a mass of fine, elongated fibres, which are matted and interlaced in a manner which very much resembles hair. In gold this fibrous structure, although present, is far less marked. The influence of extreme pressure upon gold and silver seems to be, therefore, to develope a definite internal structure. Gold and silver, in fact, appear to behave in some respects like plastic bodies. When forced to spread out in the direction of least their resistance molecules do not move uniformly, but neighboring molecules, having different velocities, glide over one another, causing a pronounced arrangement of particles in straight lines.

This development of a fibrous structure, by means of pressure, in a homogeneous substance like silver, is an interesting lesson in experimental geology, which may serve to illustrate the probable origin of the fibrous structure of the comparatively homogeneous limestones of the Pyrenees, Scotland, and the Tyrol.—*Nature.*

